# Genome Sequences of 10 SARS-CoV-2 Viral Strains Obtained by Nanopore Sequencing of Nasopharyngeal Swabs in Malta

**DOI:** 10.1128/MRA.01375-20

**Published:** 2021-01-28

**Authors:** Manuele Biazzo, Silvia Madeddu, Elfath Elnifro, Tessabella Sultana, Josie Muscat, Christian A. Scerri, Francesco Santoro, David Pinzauti

**Affiliations:** a The BioArte Limited, San Gwann, Malta; b Medical Laboratory Services within St James Hospital, Sliema, Malta; c Department of Physiology and Biochemistry, University of Malta, Msida, Malta; d Department of Medical Biotechnologies, University of Siena, Siena, Italy; DOE Joint Genome Institute

## Abstract

The genome sequences of 10 severe acute respiratory syndrome coronavirus 2 (SARS-CoV-2) strains from Sliema, Malta, were obtained by Nanopore sequencing using the amplicon sequencing approach developed by the Artic Network. The assembled genomes were analyzed with Pangolin software and assigned to the B.1 lineage, which is widely circulating in Europe.

## ANNOUNCEMENT

We present the genome sequences of 10 severe acute respiratory syndrome coronavirus 2 (SARS-CoV-2; genus *Betacoronavirus*, family *Coronaviridae*) viral strains retrieved from Sliema, Malta. Because of a relatively high incidence of SARS-CoV-2 infection in Malta, we decided to sequence these viral strains to contribute to the local and global epidemiology of SARS-CoV-2. Rapid sequencing is important for surveillance of circulating viral strains and for prompt contact tracing (1). Viral RNA was obtained from nasopharyngeal swabs collected at the St. James Hospital (Sliema, Malta) in August and September 2020 (Table 1). The research described in this work was performed in adherence to the Declaration of Helsinki; no specific authorization was issued by the University of Malta Institutional Review Board (IRB), since samples were anonymized and no human data were used. Swabs were placed in 3 ml ImproViral viral preservative medium (VPM) (Improve Medical). Viral RNA was extracted with a MagCore viral nucleic acid extraction kit (RBC Bioscience; catalog number MVN400-04) using a MagCore Plus II automated nucleic acid extractor (RBC Bioscience) and was then tested by quantitative PCR (qPCR) to assess the presence of SARS-CoV-2 RNA using the GrandPerformance SARS-CoV-2 kit (TATAA Biocenter, Sweden) targeting the RNA-dependent RNA polymerase gene. Viral RNA from qPCR-positive samples was sequenced using the multiplex PCR amplicon sequencing approach developed by the ARTIC Network (https://www.protocols.io/view/ncov-2019-sequencing-protocol-v2-bdp7i5rn?version_warning=no&step=16.3). Primers for 98 overlapping amplicons were used in two multiplex PCRs to amplify the whole viral genome, except for the conserved 5′ and 3′ untranscribed regions (UTRs). The sequencing library was prepared using the native barcode kit EXP-NBD104 from Oxford Nanopore Technologies (ONT) and sequenced on an R9.4 flow cell using a MinION MK1B device (ONT). The sequencing run was managed with MinKNOW v19.12.5, disabling live base calling. Raw Nanopore reads were base called using the GridION Guppy module v4.0.11, enabling the high-accuracy mode. Base-called reads were analyzed following the ARTIC Network pipeline (https://artic.network/ncov-2019/ncov2019-bioinformatics-sop.html). All tools were run using default parameters unless otherwise specified. In total, 2,645,399 reads were obtained (mean, 264,540; range, 73,135 to 468,322 per sample).

**TABLE 1:** Data for each Malta/Sliema SARS-CoV-2 isolate

Strain name	Sampling date (day/mo/yr)	Symptoms^*a*^	qPCR threshold cycle^*b*^	Genome size (no. of bases) /coverage (%)	GC content (%)	Gapped regions^*c*^	No. of sequenced reads	Avg sequencing depth (×)	GenBank accession no.	SRA accession no.
Malta/Sliema_1/2020	19/08/2020	NA	22.4	29,782/99.6	37.98	1–54, 29837–29903	451,721	5,700	MW079418	SRR12778135
Malta/Sliema_2/2020	04/09/2020	−	29.5	29,782/99.6	37.98	1–54, 29837–29903	381,291	4,800	MW079419	SRR12778134
Malta/Sliema_3/2020	14/08/2020	+	33.3	29,782/94	37.86	1–54, 1313–1596, 19276–19576, 21147–21387, 27512–28105, 28757–29007, 28757–29007, 29837–29903	178,878	2,200	MW079420	SRR12778133
Malta/Sliema_4/2020	07/09/2020	−	26.6	29,782/99.6	37.98	1–54, 29837–29903	387,947	4,900	MW079421	SRR12778132
Malta/Sliema_5/2020	20/08/2020	−	21.6	29,782/99.6	37.98	1–54, 29837–29903	468,322	5,900	MW079422	SRR12778131
Malta/Sliema_6/2020	07/09/2020	−	23.7	29,782/98.6	37.96	1–54, 16187–16487, 29837–29903	146,139	2,500	MW079423	SRR12789615
Malta/Sliema_7/2020	05/09/2020	NA	25.6	29,782/95.6	37.94	1–54, 2248–2851, 5260–5287, 16187–16444, 16805–17087, 29837–29903	199,902	2,900	MW079424	SRR12789614
Malta/Sliema_8/2020	07/09/2020	+	23.5	29,611/93.5	37.92	1–54, 7672–7968, 8636–8913, 9558–9806, 16187–16444, 17431–17697, 24146–24416, 29666–29903	73,135	1,200	MW079425	SRR12789613
Malta/Sliema_9/2020	07/09/2020	+	24.3	29,611/98.3	37.92	1–54, 12235–12439, 29666–29903	173,524	2,800	MW079426	SRR12789612
Malta/Sliema_10/2020	07/09/2020	+	25.2	29,782/89.4	38.06	1–54, 2432–2850, 4427–4658, 5260–5287, 7390–7651, 8636–8913, 9246–9806, 17431–17706, 20119–20200, 23833–24721, 29837–29903	184,540	2,200	MW079427	SRR12789611

a +, present; −, absent; NA, information not available.

b Obtained with the GrandPerformance SARS-CoV-2 kit (TATAA Biocenter, Sweden), targeting the RdRP gene.

c With reference to the Wuhan Hu-1 genome. All samples miss regions 1 to 54 and 29837 to 29903, which are not covered by the primers.

**FIG 1:**
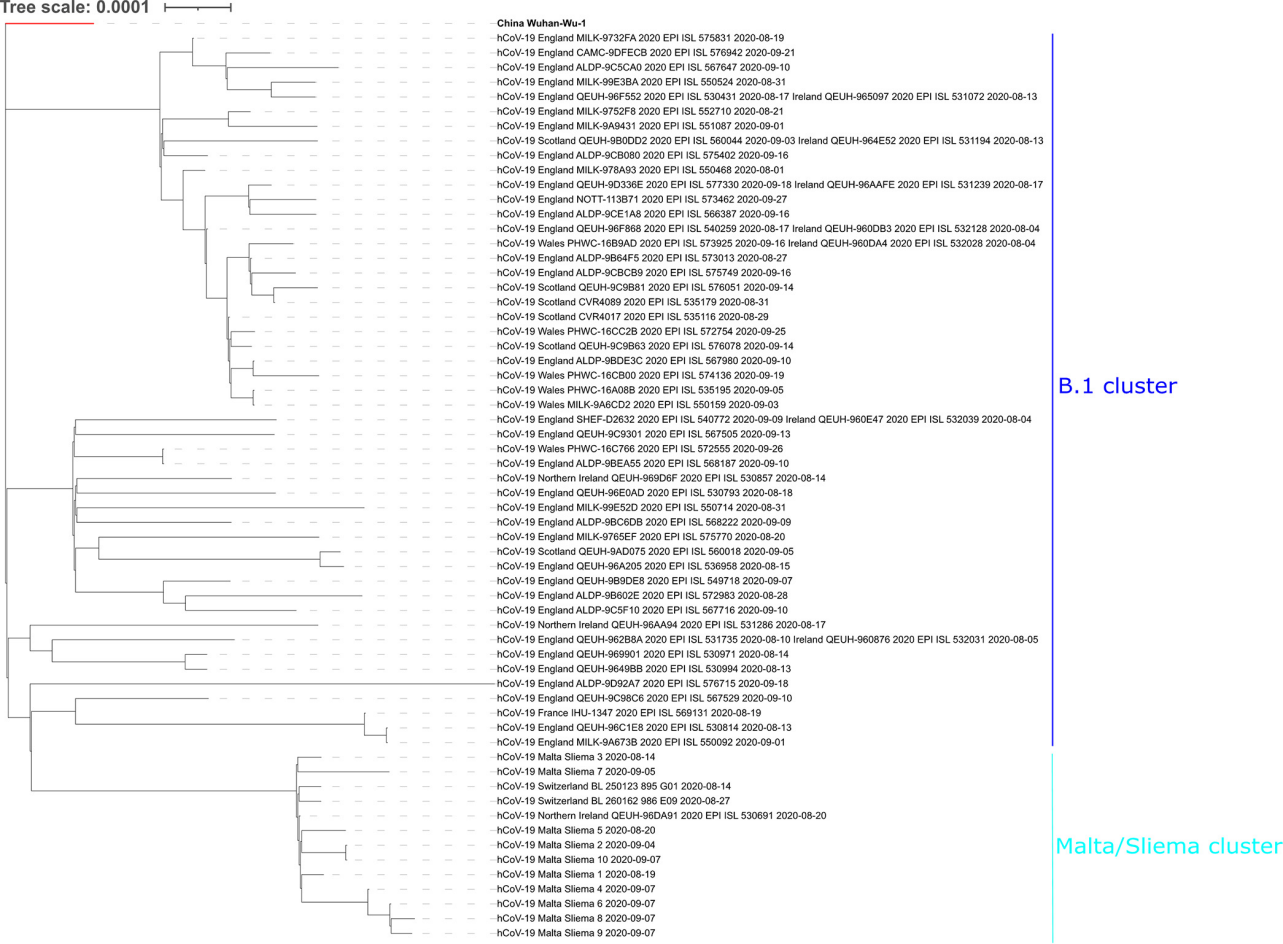
Phylogenetic analysis of Malta/Sliema SARS-CoV-2 genomes. The phylogenetic tree was generated using IQ-TREE v1.6.12 and visualized using iTOL (6). A total of 63 viral genomes are displayed, including (i) 10 genomes from Malta, (ii) 2 related genomes, Switzerland BL 2501123 and BL 2601162, (iii) 50 genomes from GISAID randomly selected among samples obtained in Europe in August and September 2020, and (iv) the SARS-CoV-2 reference Wuhan Hu-1 genome (red branch) as an outgroup. All genomes except Hu-1 belong to the B.1 cluster (blue line). The Malta/Sliema genomes cluster together with Switzerland BL 2501123 and BL 2601162 and Northern Ireland QEUH-96DA9 (light blue line), since they share the 15 nucleotide variations reported in the text. For each viral genome, the strain name and place and date of sampling are indicated.

Table 1 summarizes the information for each sample, including genome coverage and accession numbers of raw and assembled data. Four out of 10 strains (Sliema_1, Sliema_2, Sliema_4, and Sliema_5) were assembled into a near-complete genome. The six remaining samples presented gaps (from 1.7 to 10.6% of the total genome length) in the assembled sequences, associated with low or absent amplicon coverage. Compared to the reference Wuhan Hu-1 genome, a set of 15 nucleotide variations was identified, shared among all Malta/Sliema strains. Single nucleotide variations were at positions 241, 2416, 3037, 8371, 9430, 14408, 15477, 18395, 23403, 23730, 25563, 26319, 28854, and 29044, and a mutation of 3 consecutive nucleotides (AGA → TTT) was at position 20622. Variations were confirmed by visual inspection using Tablet (2). Phylogenetic analysis with the Pangolin tool v2.0.8 (3) assigned the Malta/Sliema samples to lineage B1. IQ-TREE v1.6.12 (4) was used to reconstruct a phylogenetic tree as follows: a set of representative SARS-CoV-2 genomes was downloaded from the GISAID database (https://www.gisaid.org/), including samples (i) isolated in Europe, (ii) assigned to the B1 lineage, and (iii) collected between August 1 and September 30. To date (13 October 2020), a total of 14,990 sequences have been downloaded. A total of 50 representative genomes were randomly selected using the tool seqtk (v 1.3) (https://github.com/lh3/seqtk). The samples Switzerland/BL/250123_895_G01/2020-08-14 and Switzerland/BL/260162_986_E09/2020-08-27 were added to the phylogenetic analysis, since BLAST analysis of the *Betacoronavirus* NCBI resource indicated high similarity with the Sliema genomes. Using the cat command from the Linux environment, representative genomes and Sliema samples were merged and then aligned to the reference Wuhan Hu-1 genome using the MAFFT algorithm v7.471 (5). The resulting alignment was used by IQ-TREE to reconstruct the phylogenetic tree with an ultrafast bootstrap value of 1,500 (6). The tree file was finally visualized using the Interactive Tree of Life (iTOL) Web tool (7) (Fig. 1). The Malta genomes all belonged to a separate subcluster, indicating that viral strains circulating in Malta in September 2020 were genetically homogenous. Genomic epidemiology is essential for monitoring the emergence and spread of SARS-CoV-2 variants.

### Data availability.

The Nanopore reads and genome sequences of the SARS-CoV-2 Malta/Sliema isolates are publicly available. The Sequence Read Archive (SRA) and GenBank accession numbers are listed in Table 1.
